# mTOR in Human Diseases

**DOI:** 10.3390/ijms20092351

**Published:** 2019-05-11

**Authors:** Olivier Dormond

**Affiliations:** Department of Visceral Surgery Lausanne University Hospital and University of Lausanne, Pavillon 4, Av de Beaumont, 1011 Lausanne, Switzerland; Olivier.Dormond@chuv.ch

The human body regenerates constantly in part under the control of signaling pathways that regulate cell growth. Among these pathways, the mechanistic target of rapamycin (mTOR) has emerged as a major cellular crossroad that links favorable environmental conditions with cell growth. Accordingly, mTOR is implicated in different physiological and pathological conditions, and inhibition of mTOR has been approved for various clinical situations. This special issue “mTOR in human diseases” covers different aspects of the implication of mTOR in physiological processes as well as in various diseases.

The role of mTOR and the consequences of mTOR inhibition has been extensively explored in cancer. Tian et al. review mTOR signaling in solid malignancies and discuss results of clinical trials that have tested mTOR inhibitors in eight different tumors, including lung, colorectal, gastric, renal, bladder, prostate and breast cancers as well as head and neck squamous cell carcinoma [1]. The rationale to target mTOR in advanced biliary tract cancers and in medulloblastoma is also presented by Wu et al. and Aldaregia et al., respectively [2,3]. Besides solid tumors, two reviews highlight the role of mTOR signaling in leukemia and particularly in T-cell acute lymphoblastic leukemia and provide future perspective regarding mTOR-targeting agents [4,5]. All together, these reviews acknowledge the participation of mTOR signaling pathway in tumorigenesis but also highlight the lack of major anti-tumor efficacy of mTOR inhibitors in patients. Limitations include activation of alternate proliferative signaling pathways following mTOR inhibition, tumor heterogeneity and treatment-resistant mTOR mutations. Hence, additional studies are needed to further understand the role of mTOR signaling pathway in cancer and to characterize resistance mechanisms developed by cancer cells to bypass mTOR inhibition. In this context, Tavares et al. present the contribution of mTORC1 and mTORC2 in papillary thyroid carcinoma [6]. Hsu et al. provide results on mTOR in oral cavity squamous cell carcinoma and show the anti-cancer efficacy of the dual PI3K/mTOR inhibitor NVP-BEZ235 [7]. Harachi et al. describe the importance of mTORC1 and mTORC2 in cancer cell metabolism [8]. Identification of biomarkers that predict response to mTOR inhibitors will further help improve the anti-cancer efficacy of these inhibitors. Nepstad et al. found metabolic differences in human acute myeloid leukemia cells between responders and non-responders to mTOR inhibition [9]. Whereas next-generation sequencing is a valuable tool to identify biomarkers, Seeboeck et al. demonstrate, however, that commercially available ready-made gene panels show limited applicability for mTOR pathway-related genes [10]. Besides cancer cells, mTOR signaling pathway regulates cellular processes of non-tumorous cells present in the tumor microenvironment, such as endothelial cells, lymphocytes and macrophages. Conciatori et al. review the role of mTOR in these cells and highlight the anti-cancer benefits that result from mTOR inhibition in the microenvironment [11]. Finally, tumor cachexia is associated with poor prognosis in cancer patients. Emerging evidence suggests that mTOR influences cachexia, as discussed by Duval et al. [12].

Besides cancer, the implication of mTOR signaling pathway in neurological and neuropsychiatric disorders has been demonstrated. Ryskalin et al. present evidence that autophagy impairment is involved in synaptic dysfunction found in some psychiatric disorders, such as schizophrenia. Accordingly, mTOR inhibitors that induce autophagy might represent a therapeutic intervention [13]. Similarly, accelerating autophagic flux appears to be an effective treatment strategy in Parkinson’s and Alzheimer’s diseases and two reviews present the role of mTOR and the therapeutic opportunities for mTOR inhibitors in these diseases [14,15]. Neurodegenerative diseases are also part of age-related pathologies. Interestingly, recent studies have highlighted mTOR inhibitors as promising treatment for various age-related disorders and are discussed by Walters and Cox [16]. mTOR is further involved in Hutchinson–Gilford progeria syndrome, a rare premature ageing syndrome. Chiarini et al. provide a complete review on the role of mTOR in this disease as well as in other laminopathies and discuss therapeutic opportunities for mTOR inhibitors [17].

Several side effects have been observed in patients treated with mTOR inhibitors. In particular, lung toxicity such as lung fibrosis results in frequent therapy discontinuation. Granata et al. performed mRNA and microRNA profiling on primary bronchial epithelial cells treated or not treated with mTOR inhibitors, which led to the identification of novel potential targets [18]. mTOR inhibitors also reduce male fertility, and the mechanisms controlled by mTOR in the male reproductive tract are presented by Moreira et al. [19]. Toxicities mediated by drugs might also involve mTOR activation. For instance, general anesthetic agents harm brain development. Xu et al. suggest that anesthetic agents-mediated neuron disruption involves upregulation of mTOR activity [20].

Over the last decade, multiple studies have unveiled the complex role played by mTOR signaling pathway in cellular metabolism. Mao and Zhang discuss recent findings on the role of mTOR signaling pathway in metabolic tissues and organs including liver, adipose tissue, muscle and pancreas [21]. Sangüesa et al. highlight the consequences of mTOR activation by excessive consumption of sugar [22]. In addition to cellular metabolism, mTOR regulates autophagy. Wang et al. show that mTOR participates in dopamine receptor D3-mediated autophagy regulation [23]. Finally, Kim et al. found mTOR pathway activation by fluid shear stress and melatonin in preosteoblast cells [24]. 

In summary, this special issue highlights the fascinating role played by mTOR in cellular processes. It further addresses a non-exhaustive panel of human diseases in which mTOR is implicated, from rare disorders to cancer.
